# Centeredness Theory: Understanding and Measuring Well-Being Across Core Life Domains

**DOI:** 10.3389/fpsyg.2018.00610

**Published:** 2018-05-01

**Authors:** Zephyr T. Bloch-Jorgensen, Patrick J. Cilione, William W. H. Yeung, Justine M. Gatt

**Affiliations:** ^1^MAP Corp. Pte. Ltd., Atlanta, Atlanta, GA, United States; ^2^MAP Corp. Pte. Ltd., Singapore, Singapore; ^3^Sciens Pty. Ltd., Bundoora, VIC, Australia; ^4^School of Psychology, University of New South Wales, Sydney, NSW, Australia; ^5^Neuroscience Research Australia, Randwick, NSW, Australia

**Keywords:** wellbeing, flourishing, mental health, mindfulness, self-actualization, goal-setting, COMPAS-W

## Abstract

**Background:** Centeredness Theory (CT) is proposed as a new mental health paradigm that focuses on well-being at a systems-level, across the core life domains of the self, the family unit, relationships, community, and work. The current studies aimed to validate the psychometric properties of a new scale that measures CT against existing well-being and mental health measures.

**Methods:** Study 1 included 488 anonymous online respondents (46% females, 28% males, 25% unknown with median age between 31 and 35 years) across 38 countries who completed the CT scale. Study 2 included 49 first-year psychology students (90% females, mean age of 19 years) from Sydney Australia that completed the CT scale and other well-being and mental health questionnaires at baseline and 2-weeks follow-up.

**Results:** Exploratory and confirmatory factor analyses resulted in a refined 60-item CT scale with five domains, each with four sub-domains. The CT scale demonstrated good internal consistency reliability and test-retest reliability, and showed evidence of convergent validity against other well-being measures (e.g., COMPAS-W Wellbeing Scale, SWLS scale, and Ryff's Psychological Well-being scale).

**Conclusions:** The CT scale appears to be a reliable measure of well-being at a systems-level. Future studies need to confirm these findings in larger heterogeneous samples.

## Introduction

A shift is underway in psychiatric research to understand mental health and well-being as more than just the risk factors for mental illness alone (Maddux, 2008). Optimal well-being spans superior psychological and physiological functioning and has been shown to predict increased longevity and healthy aging (Seligman, 2008; Diener and Chan, 2011), resistance to infection (Cohen et al., 2006; Steptoe et al., 2009), and reduced risk for illness and mortality (Danner et al., 2001; Kubzansky and Thurston, 2007; Chida and Steptoe, 2008). However, the science of well-being remains at an early stage, with particularly little known about the upper end of the well-being spectrum (Huppert and So, 2013). If measures can be designed and tested to identify the mechanisms that foster optimal well-being across the core domains of life, then better interventions for public health can be developed.

### Current models of well-being

Well-being is currently defined by three paradigms: Subjective well-being, psychological well-being, and composite well-being. Subjective well-being (or *Hedonia*) defines and measures positive and negative affect, and satisfaction with life (Diener et al., 1985, 2010). It is based on the notion that increased pleasure and decreased pain leads to happiness (Carruthers and Hood, 2004). Psychological well-being (or *Eudaimonia*) defines and measures attributes such as autonomy, positive relations with others, life purpose, mastery, and personal growth (Ryff, 1989; Ryff and Keyes, 1995). It is based on the notion that well-being is the feeling that accompanies behavior in the direction of, and consistent with, one's true potential (Waterman, 1984). Lastly, composite well-being, as the name intimates, views subjective well-being and psychological well-being as conceptually related but distinct streams of positive psychological functioning, and accumulating evidence highlights the merits in measuring both components using composite indices such as the Mental Health Continuum (MHC-LF) (Keyes, 2007) or the COMPAS-W Wellbeing Scale (Gatt et al., 2014). Moreover, evidence from twin studies suggests that common genetic factors contribute to composite well-being and its subjective and psychological subcomponents (Keyes et al., 2010; Kendler et al., 2011; Gatt et al., 2014), and that measures of composite well-being are related but conceptually distinct from mental illness measures such as anxiety and depression symptoms (Routledge et al., 2016).

### Centeredness theory (CT)

**Figure 1:**
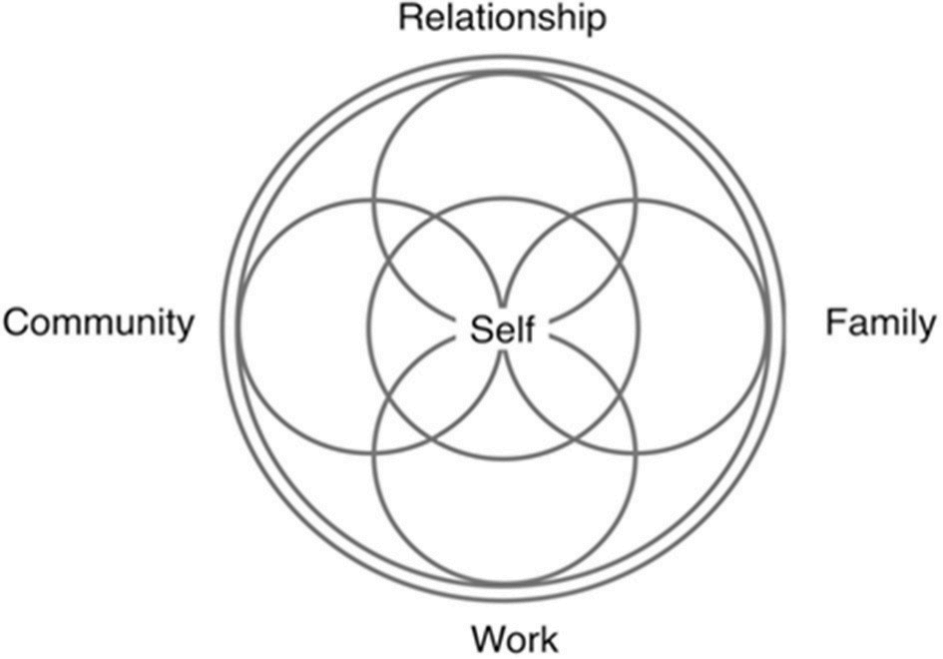
A schematic of Centeredness Theory's five geometric domains.

Building on these paradigms, it may also be important to consider well-being at a systems-level; that is, across the core domains of life, spanning the self, relationships, the family unit, community, and work. A new paradigm is Centeredness Theory (CT) which considers well-being using a systems approach to “self-actualization” (the realization or fulfillment of one's talents and potentialities) across the five life domains of Self, Relationship, Family, Work, and Community (Bloch-Jorgensen, 2015). These five domains can be visualized as a geometric pattern of five spheres with a central middle sphere measuring Self (an endogenous internal measure), surrounded equidistantly by four other spheres measuring Relationship, Family, Work, and Community (co-existing exogenous measures, external to the Self) as shown in schematic form in Figure 1.

CT applies a heuristic model of mental balance (Wallace and Shapiro, 2006) and is founded on an open systems perspective that suggests that the five life domains are interconnected, and that the balance (or imbalance) within one domain facilitates (or inhibits) balance in the other domains. The theoretical framework of CT is based on the homeostatic need to achieve balance between one's inner endogenous state and outer exogenous environment. To promote balance both between domains (that is, between the Self and the exogenous domains) and within domains (that is, between sub-domains), intrinsic self-generated goals (Kasser and Ryan, 1996) must be implemented that are approach-orientated (Elliot et al., 1997) and congruent with one's personal values (Sheldon and Kasser, 1998). Centeredness is achieved when meaningful goals exist in all five domains and when balance is achieved within and between the five domains through congruent thought and behavior. With centeredness, each domain can be mastered and expanded. Awareness, thought and habit determine the quality of goals and actions, and ultimately, the ability to self-actualize and increase well-being.

This geometric open system model is reminiscent of Ryff's spiral of mind-body influence (Ryff, 1989) and Fredrickson's broaden-and-build theory: positive emotions trigger upward cognitive spirals that broaden thought-action repertoires which increase well-being, whereas negative emotions narrow thought-action repertoires to a state of fight or flight (Fredrickson and Joiner, 2002). CT hypothesizes that each thought and action made within the system influences thoughts and actions both *within* and *between* the domains, which allows for interaction and bidirectional feedback (Nowak and Vallacher, 1998). The system is regulated by the ability to decipher and master chaos within the self and in response to the exogenous environments. Optimal regulation of the five domains is underpinned by the ability to regulate chaos with a neurological, physiological and behavioral repertoire that is adaptable and flexible to everyday challenges (Goldberger et al., 1990; Freeman, 1991). Over time, it is predicted that the ability to regulate unpredictability and change in the domains yields resilience; an inherent ability to adapt and maintain optimal well-being despite exposure to trauma and adversity (American Psychological Association, 2010).

To enhance such a system, a fit must be created and maintained between the self and the exogenous environments that complement intrinsic goals. The characteristics of such a fit would be reflected by the enhanced ability to reframe stress, to be mindful, and to cultivate awareness and formulate meaningful goals. By using these skills together, one can self-actualize and thereby achieve higher states of well-being.

#### Reframe stress

CT proposes that the ability to reframe debilitative stress into enhancive stress is a characteristic of centeredness. A “stress-is-enhancing” mind-set interprets stress as a driver to achieve beneficial outcomes, whereas a “stress-is-debilitating” mind-set experiences the stressor as a hindrance to health (Crum et al., 2013). With the enhancive state, the stressor can be accepted and used to achieve a positive outcome. In contrast, the debilitative state either incorporates avoidance strategies or it mismanages the stressor and its consequential emotional or physiological impact (Crum et al., 2013). In this sense, CT takes into account the degree of awareness around the stressor (e.g., the fact that a relationship is in trouble) and the degree to which self-awareness and goals have been formulated to master it. Moreover, CT identifies the degree to which self-awareness operates inside each of the five life domains in terms of perspective-taking, vulnerability, empathy, and forgiveness. It is therefore the goal of the individual to close the gap between the current state of well-being and the ideal.

#### Mindfulness and reflection

An aspiration-centered self, elemental to CT, is facilitated by mindfulness because it is a state conducive to create and adhere to meaningful goals. Mindfulness is a type of awareness where attention is placed continuously on a familiar object or state without distraction (Asanga, 2001). This attention is directed singularly to present sensations and perceptions (e.g., through questions such as, “What do I see right now?” and “What do I hear right now?”) to transcend rumination and unwanted thoughts. Mindfulness can also support meta-attention: the ability to monitor the state of mind, and recognize when attention or emotion has succumbed to hyper- or hypo-activity (Nanamoli and Bodhi, 1995). This is helpful to regulate emotion and to foster a metacognitive attentional state that is non-reactive, non-evaluative, and can monitor moment-by-moment cognition, emotion, perception, and sensation without thoughts being fixed on past or future (Garland, 2007; Lutz et al., 2008). Evidence suggests that mindfulness and mindful learning (Langer, 2000) promote emotional well-being (Brown and Ryan, 2003; Shapiro et al., 2008) because they reduce ruminative thinking and negative affect (Frewen et al., 2008) and foster acceptance (Shapiro et al., 2006). Mindfulness is therefore instrumental to CT because it emphasizes awareness of the present, characterizes an open and receptive processing of events, and frames an internal environment that is conducive to create and actualize meaningful goals across the five life domains.

#### Meaningful goals

Goals play an essential part in CT because a goal that is intentional and active is integral to achieve change and to influence further goal striving (van Dierendonck et al., 2009) and mental balance. Extensive research highlights the core elements that are required to optimize well-being through the pursuit of goals. Firstly, goals need to be intrinsic, or “self-generated”; they need to satisfy basic psychological needs and to not be contingent on the reactions of others (Kasser and Ryan, 1996). Some examples include self-acceptance, growth and autonomy, having satisfying relationships with others, community activism, and physical fitness, and feeling healthy. In contrast, extrinsic goals, associated with reduced well-being are a means to an end, contingent on others and include pursuits such as achieving financial success, social recognition and being admired, and looking attractive (Kasser and Ryan, 1996). Indeed, the structure of intrinsic goals is common across all people, regardless of cultural differences (Grouzet et al., 2005). Secondly, higher well-being is also associated with the self-regulated pursuit of approach goals, and the movement away from avoidance goals. Approach goals are focused on a positive outcome or state (e.g., to be open and cheerful when meeting new people, to take on leadership roles at work, or to exercise regularly for improved fitness), whereas avoidance goals are focused on moving away from a negative outcome or state (e.g., to stop being boring at parties, to not become a follower at work, or to try and avoid eating fast foods) (Elliot et al., 1997). Thirdly, a goal is more likely to be realized in the presence of strong social and self-regulatory skills, where there is a general positive belief in the goal, and where the goal is congruent with inherent psychological needs (Sheldon and Kasser, 1998). These needs depend on self-concept and self-related wishes, as well as the demands inherent in the environment (Brunstein et al., 1998). Therefore, if a set of motives are oriented toward the achievement of independence, self-assertion, and mastery, then goals that are incongruent to this will lead to reduced well-being, and the converse if aligned (Brunstein et al., 1998).

In selecting goals, some individuals make the mistake of choosing goals that are not representative of the values of their “self.” This occurs when individuals select goals that are not representative of their authentic interests and values and are limited by the dictates of others (i.e., “should” and “ought”) and their own anxiety, fear, or guilt in not fulfilling true desires. If these extrinsic “should” goals are pursued, the strength of such goals is weakened when obstacles are encountered because they are not congruent with the belief system of the self. In contrast, intrinsic goals that are pursued and evolve directly from self-choices have a long-term impact on elevated well-being (Sheldon and Elliot, 1999). Most individuals have multiple goals. Some goals may conflict, like the conflict between professional aspirations and the aspirations for family, and lead to cognitive dissonance. CT hypothesizes that centeredness is facilitated when meaningful goals are present in all five domains.

### The centeredness theory (CT) scale

The Centeredness Theory (CT) Scale was designed to measure CT; that is, centeredness across the five life domains of Self, Family, Relationship, Work, and Community, as shown in Figure 1. A key purpose of the scale is to measure the degree to which meaningful goals, mindfulness, and the ability to reframe stress operates within and between the five domains (and sub-domains). It was designed from content previously developed (Bloch-Jorgensen, 2015). Items were developed to assess each domain (Supplementary Figure 1) and sub-domain (Supplementary Figure 2). Each domain contained four sub-domains: (1) Self: Adaptability, Awareness, Contentment, and Inspiration; (2) Family: Participation, Communication, Care, and Receptiveness; (3) Relationship: Connection, Attentiveness, Understanding, and Enrichment; (4) Work: Engagement, Innovation, Accountability, Supportiveness; and (5) Community: Sympathy, Sensitivity, Empathy, and Confidence. Questions were developed to measure the core features of the domain and its subcomponents; for instance, for the Self domain, example items included “Do you welcome change?” (Adaptability), “Do you ever acknowledge your fear?” (Awareness), “Do you feel satisfied with who you are?” (Contentment), and “Do you think about your life and what you can do to make it more rewarding?” (Inspiration).

A multi-disciplinary set of theories and disciplines contributed to the formation of CT including but not limited to systems theory (Laszlo, 1996; Senge, 2006), the productive personality orientation (Fromm, 1947), self-actualization (Maslow, 1954), individuation (Jung, 1980), flow (Cziksenmihalyi, 2008), chaos theory and fractal geometry (Mandelbrot, 1983; Peitgen et al., 1992), and catastrophe theory (Thom, 1975). Each theory informed different aspects of CT. For instance, chaos theory intimated that both within and between the life domains exists a non-linear system and a “sensitive dependence on initial conditions” (Peitgen et al., 1992). Systems Theory (Laszlo, 1996) signaled that there is a relationship within and between the domains and that an intricate interplay exists across both a hidden and patent set of dimensions. Thom's catastrophe theory (Thom, 1975) suggests that self-intensifying action is inherent in nature and, specifically, the ability to be stable is a prerequisite to create a new pathway, such as new biological pathways. A pragmatic example of this is the fortifying of optimal networks in the brain following the practice of mindfulness. From the perspective of CT, flow (Cziksenmihalyi, 2008) becomes a consequence of centeredness, as the individual learns to define and distinguish themselves from others (Jung, 1980), a focus on being optimally productive in all life domains (Fromm, 1947) develops, and as the individual self-actualizes, the interplay of life domains can broaden, learn from and influence the local and wider social network (Senge, 2006). Inherent to the scale are a number of key inferences. First, that the majority of the population would possess meaningful goals within the core domains of work, family, relationship, community and self, and this would be irrespective of cultural context (namely, collectivistic versus individualistic paradigms). Second, that while intrinsic goals may exist, the goal may vary in its level of dormancy and patency. For instance, one may foresee a goal that would make one happier yet they may lack the appropriate skill-set to actualize the goal to achieve that state (e.g., fostering a loving relationship, a meaningful career, or a cohesive family unit). One purpose behind the design of each question was to enable the individual to become more self-aware of such disparities in goals versus resources to help guide strategies needed. Third, the domain *Self* contains four sub-domains (Awareness, Contentment, Adaptability, and Inspiration) which play a core function in identifying, fostering, and actualizing meaningful goals across the four exogenous domains. In particular, *Inspiration* measures whether the goal is intrinsic; *Contentment* measures whether attainment of the goal is enjoyed; *Adaptability* measures the ability to process the environment and adaptively respond to it; and *Awareness* measures familiarity to both self and environment and an awareness of the gap between the current reality and the goal. Fourth, the domains are likely to interact and impact one another. For instance, the ability to care within the Family domain may impact the ability to be supportive in the Work domain, and vice versa.

### Aims of the current studies

The current studies aimed to validate the new CT Scale. In Study 1, a psychometric validation study of the original and refined scale was conducted using exploratory and confirmatory factor analytic techniques. These analyses aimed to confirm whether the CT domains and sub-domains were structurally sound, and to quantify their contribution to overall centeredness. In Study 2, an independent reliability study of the refined scale was conducted to confirm its internal consistency reliability, test-retest reliability, and convergent validity against existing well-being and mental health questionnaires.

## Study 1

### Methods

#### Participants and design

Participants consisted of 488 first-time anonymous online respondents across 38 countries, with the principal locations being Oceania (29.70%), North America (15.20%), United Kingdom (13.70%), Europe (8.30%), Asia (4.50%), South America (2.00%), Africa (1.20%), and 24.40% did not specify a country. Participants constituted 226 females (134 in a relationship, 84 single, 8 other), 138 males (94 in a relationship, 35 single, 9 other), and 124 who did not provide any information. Age was measured on a categorical scale from 1 to 12 (where “1” corresponds to <20 years and “12” corresponds to 70+ years, with intervals of 5 years). The median age group is between 31–35 with a lower and upper quartile range being between 21 and 50. Up to 70% of participants reported that they were employed, 29% did not state their employment and 1% were retired. Prior to completing the questionnaire, all respondents agreed to supply their responses for possible use in future scientific research, with anonymity maintained. Ethical approval was thus not required according to national guidelines which stipulate that “negligible risk research” which involves the use of “existing collections of data or records that contain only non-identifiable data about human beings” may be exempt from ethical review (National Health and Medical Research Council, 2007, p. 70, Updated May 2015).

#### Measure: the CT scale

Construction of the CT scale was initiated by writing definitions for the five domains of Centeredness (Supplementary Figure 1) and twenty sub-domains (Supplementary Figure 2). Approximately 150 questions were originally created, making up approximately 30 items per domain. The items were generated by two item writers who were asked to write self-descriptive items that fit the theory and that could be applied to men and women and adults across the lifespan. No questions were derived or adapted from existing published questionnaires. Approximately a third of items were then removed due to a number of criteria, including item ambiguity, item redundancy, or poor fit to scale descriptions. The remaining items were 108 in total, or approximately 20 items per domain (see Supplementary Table 1). One-hundred of the 108 items are measured on a Likert rating scale from 1 to 6 ranging from *Never* to *Always* (1 = “Never,” 2 = “Rarely,” 3 = “Occasionally,” 4 = “Regularly,” 5 = “Mostly,” 6 = “Always”). Eight of the 108 items were not included in the analysis because they were only added as a technique to maintain the participant's focus on completing the questionnaire (i.e., asking the participant, “Did you pause before answering that question?” with a “yes/no” response).

#### Analysis

The 488 respondents were used as the training set to create the optimal factor solution using exploratory factor analysis (EFA), and then two random samples were created to test and validate the solution: N_1_ (subsample 1) = 255, N_2_ (subsample 2) = 233. Holding with the theory, the 100 item CT scale (with the exclusion of the eight additional test items) was assessed to validate the structural integrity of the five domains separately. Several criteria were used to evaluate the optimal factorial structure for each domain. These criteria included an initial examination of the scree plot loadings, in combination with Velicer's MAP Procedure (which is based on achieving minimum BIC statistic, or Bayesian Information Criteria) and Horn's Parallel Analysis (which is based on eigenvalue cut-offs >1) (Courtney, 2013). Once the optimal factors were determined for each domain (i.e., four factors in the current study), an iterative EFA was applied to reduce the number of items per domain. Using the EFA settings of a principal components extraction method and varimax normalized factor rotation, an algorithm was used to iteratively remove items with lowest maximum loading across all factors that were below a set threshold (0.5 was the default). One item was removed at a time and the analysis was re-run. This process was applied to the five domains separately until we achieved a balanced design of 12 items and four factors (three items per factor) per domain. This approach to item reduction was set to maintain the theoretical assumptions of CT, and at the same time to minimize the item set to a number that was deemed reasonable. The EFA item-reduction procedure was conducted across the population (*N* = 488), and then the final factor solution was tested within subsample 1 (*N* = 255) and validated against subsample 2 (*N* = 233). Across the five domains, the subsample 1 and subsample 2 sets implied that there were cross loadings across three items within Family (Care and Receptiveness), one item within Self (Adaptability), one item within Relationship (Connection), two items within Work (Engagement and Accountability), and two items within Community (Empathy and Confidence). These nine items were retained in the scale.

Confirmatory factor modeling (CFM) was then used to assess the structural integrity of the five domains (Family, Self, Relationship, Work, and Community) using *LISREL 9.2*. CFM models were also employed to compute the composite scores for each of the five domains (Family, Self, Relationship, Work, and Community). The computation was obtained by fitting a one-factor congeneric measurement model (Joreskog, 1971) to the four sub-domains (as shown in Supplementary Figure 3). The domains were then re-scaled by using factor score regression indices obtained from the CFM one-factor models. This minimized the measurement error in the indicators that contributed to each composite scale, thus increasing reliability (and validity) of the computed composite scores (Holmes-Smith and Rowe, 1994). A CFM model was then employed to assess the contribution that each rescaled domain had on centeredness. The criteria used to assess the models' fit (Hooper et al., 2008) was based on the Chi-Square statistic, its degrees of freedom and *p*-value, the root mean square error of approximation (RMSEA) and its associated confidence interval, the standardized root mean square residual (SRMR), the comparative fit index (CFI) and one parsimony fit index such as the parsimonious normed fit index (PNFI). These indices have been chosen over other indices as they have been found to be the most insensitive to sample size, model misspecification, and parameter estimates (see Supplementary Table 2).

Two structural equation models were compared to test the theoretical assumptions contained in CT, namely a second-order CFM and an improved nested model (i.e., with the addition of cross-loadings between domains and sub-domains). A chi-square difference test was conducted, where the second-order CFM was treated as the baseline and then compared to the improved nested model.

### Results

#### EFA: questionnaire factor assessment and item reduction

Across the whole sample, the EFA and factor retention techniques of the original 100 items suggested that each of the five domains contained four to six factors. The domain Family achieved a minimum BIC with four factors (−306) for Velicer's Map Procedure and five factors for Horn's Parallel; Self achieved a minimum BIC with five factors (−334) for Velicer's Map Procedure and six factors for Horn's Parallel; Relationship achieved a minimum BIC with six factors (−424) for Velicer's Map Procedure and four factors for Horn's Parallel; Work achieved a minimum BIC with four factors (−518) for Velicer's Map Procedure and five factors for Horn's Parallel; and Community achieved a minimum BIC with four factors (−306) for Velicer's Map Procedure and four factors for Horn's Parallel. Together, the results indicated that a four factor solution within each domain provided the clearest structure, with the minimal optimal solution consisting of 60 items (12 per domain and 3 per sub-domain) (see Supplementary Table 3 for the EFA results for the original 100-item vs. final 60-item solution in the full sample). Each domain accounted for 64.79 to 80.88% of total variance (Family 75.51%), Self (70.92%), Relationship (80.88%), Work (69.23%), and Community (64.79%).

The final factor model was independently confirmed in the subsample 1 (test) and subsample 2 (validation) sets, checking for the same optimal factor solution and item loadings. The resulting factor loadings, total variance, and Cronbach alphas for test and validation are provided in Supplementary Table 4. Overall, we found that the majority of items met the minimum factor loading threshold (>0.5) in one or both data sets. A few items met this threshold in one sample, but estimates were weaker in the second sample (i.e., items 2, 3, and 10 for Family, item 13 in Self, item 41 in Work, and items 51, 56, and 60 in Community). Moreover, item 33 in Relationship loaded only ~0.30 in both samples (see Supplementary Table 4). The internal consistency reliability of each sub-domain ranged from moderate (0.63 for the Community “Sensitivity” sub-domain in subsample 1, and 0.67 for the Work “Engagement” sub-domain in subsample 2) to excellent (0.92 for the Relationship “Understanding” sub-domain in both subsamples 1 and 2); with the average internal consistency reliability of each domain being: Family (0.81, subsample 1; 0.84, subsample 2), Self (0.78, subsample 1; 0.78, subsample 2), Relationship (0.88, subsample 1; 0.84, subsample 2), Work (0.73, subsample 1; 0.73, subsample 2), and Community (0.68, subsample 1; 0.72, subsample 2). The inter-correlations between the domains in samples 1 and 2 were all positive and significant (see Supplementary Table 7).

#### CFM: structural integrity of the domains

**Figure 2:**
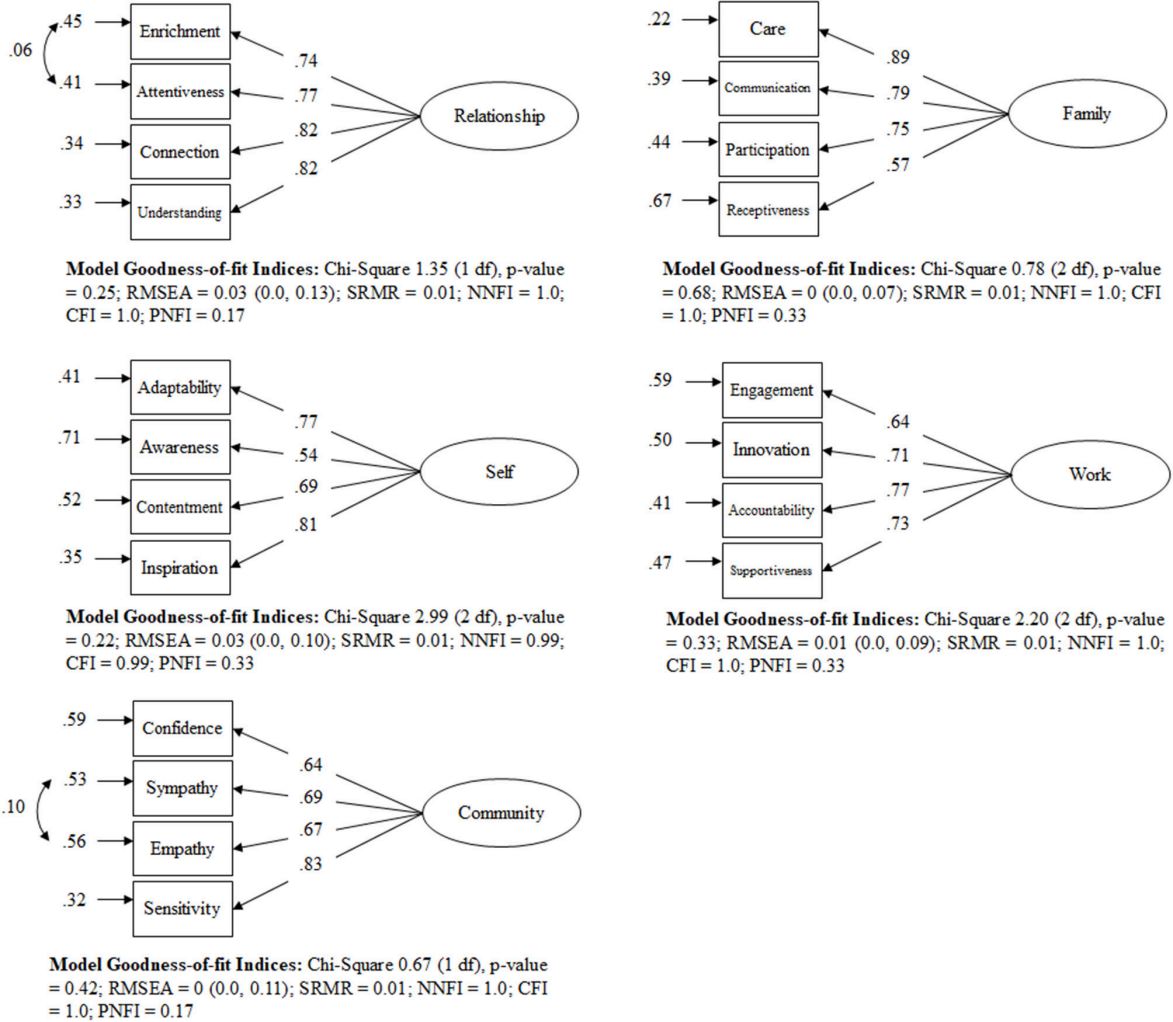
Standardized solution of the one-factor models for each domain of the CT Scale.

Figure 2 displays the congeneric models fitted to each composite scale, including the model goodness-of-fit (GOF) indices. The models' criteria demonstrates that each domain showed an excellent fit to each of their four sub-domains, and that each model accounted for >99% of the relative variances and co-variances, confirming the stability of each domain.

Supplementary Table 5 provides the item weights (factor score coefficients) and Cronbach alphas in the total sample. All Cronbach alphas have a reliability measure >0.70, which meets the stated criteria for good internal consistency reliability (Tavakol and Dennick, 2011). This table also provides the respective factor score coefficients for each CFM, with their respective proportionally weighted factor score coefficients (Rowe, 2006). As all weighted factor score coefficients sum up to 1, new domain scores can be obtained maintaining the original scale. The respective formulas for each domain are:

$$\begin{array}{r} Family\;=\;fa1^{*}0.481\;+\;fa2^{*}0.207\;+\;fa3^{*}0.190+fa4^{*}0.122\\ Self\;=\;se1^{*}0.337\;+\;se2^{*}0.124\;+\;se3^{*}0.194+se4^{*}0.345\\ Relationship\;=\;re1^{*}0.163\;+\;re2^{*}0.240\;+\;re3^{*}0.280+re4^{*}0.317\\ Work\;=\;wo1^{*}0.155\;+\;wo2^{*}0.235\;+\;wo3^{*}0.299+wo4^{*}0.311\\ Community\;=\;ca1^{*}0.174\;+\;ca2^{*}0.219\;+\;ca3^{*}0.175+ca4^{*}0.432 \end{array}$$

#### Measuring centeredness

**Figure 3:**
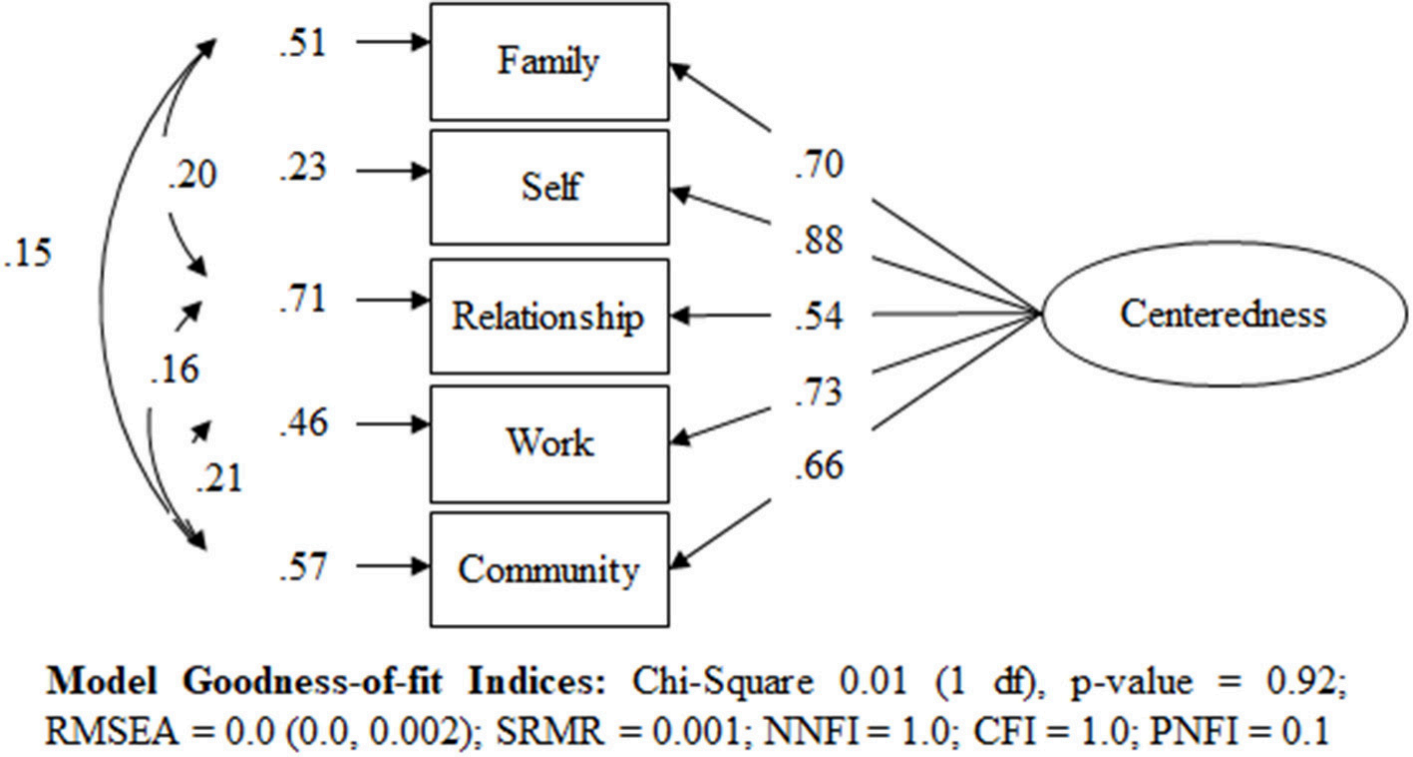
Standardized solution of the one-factor model for Centeredness using the CT Scale.

Supplementary Figure 4 shows how the proportionally weighted scores for each domain are applied to measure centeredness by applying a one-factor congeneric measurement model. The one-factor congeneric measurement model measures centeredness based on the endogenous and exogenous factors where the exogenous domain factors of Family, Relationship, Work, and Community error variances are correlated (note: to avoid the model being saturated not all co-variances are correlated). The models' criteria demonstrates that centeredness has an excellent fit to each of their five weighted domains, accounting for >99% of the relative variances and co-variances in the data (Figure 3). The weighted factor score coefficients illustrate that the inner domain—Self—accounts for 54.4% (based on proportionally weighted score coefficient) and the combined scores for the outer domains account for 46.6% as shown in Supplementary Table 6.

#### Testing the structural theoretical model for centeredness theory

**Figure 4:**
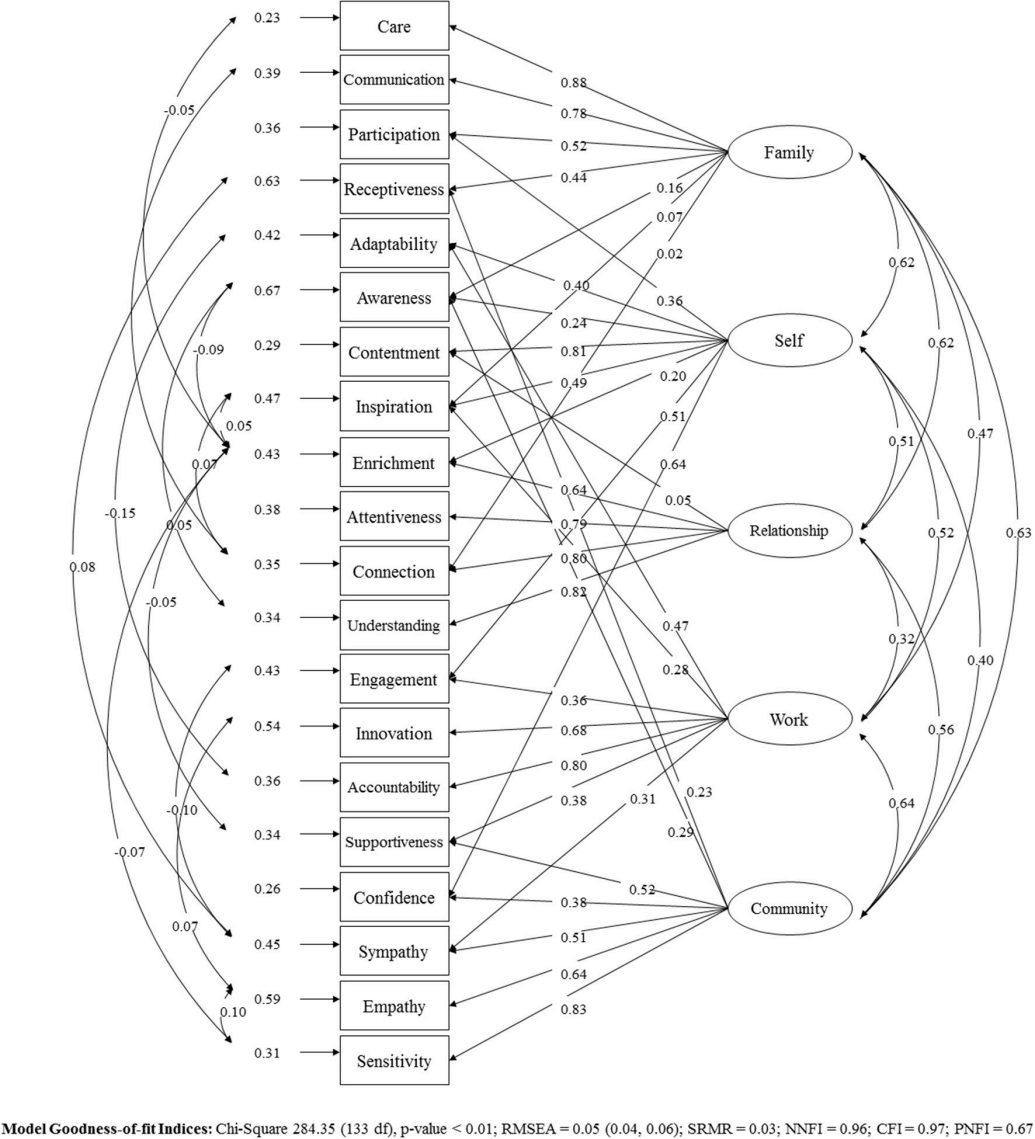
Standardized solution of the structural model to measure Centeredness Theory.

In order to take into account the domains and sub-domains, two alternative models were compared to measure centeredness: (1) a second-order CFM (which assumed that the sub-domains load directly onto their respective domains only) and (2) an improved nested model (which tested the inclusion of cross-loadings from sub-domains to other domains). The Chi-square difference test (459.71, *df* = 14, *p* < 0.001) indicated that the nested model (Chi-square = 284.35, *df* = 133, *p* < 0.001, RMSEA = 0.048 (0.041, 0.056), SRMR = 0.028, NNFI = 0.961, CFI = 0.973, PNFI = 0.665) was a significant improvement from the second-order CFM model (Chi-square = 744.06, *df* = 147, *p* < 0.001, RMSEA = 0.091 (0.085, 0.098), SRMR = 0.064, NNFI = 0.860, CFI = 0.892, PNFI = 0.672) (see Figure 4). Although the chi-square *p*-value was below 0.05, all other GOF indices were acceptable and so to minimize data over-fitting, we decided the nested model was the best model representing CT.

#### Sample demographics

The means and standard deviations (SD) of subsamples 1 and 2 for the CT Total Well-being scale and domain scores are provided in Table 1.

**Table 1:** Sample means (± Standard Deviations) for the CT scales in Study 1.

**Measure**	**CT total well-being**	**CT family**	**CT self**	**CT relationship**	**CT work**	**CT community**
Study 1 (subsample 1, *N* = 255)	72.97 ± 12.64	71.31 ± 14.13	65.14 ± 18.31	73.33 ± 15.46	76.70 ± 12.89	78.39 ± 10.35
Study 1 (subsample 2, *N* = 233)	72.35 ± 12.76	70.37 ± 14.77	64.31 ± 18.08	74.15 ± 14.41	75.71 ± 13.23	77.20 ± 11.28

*Study 1 included 488 anonymous online adult respondents across 38 countries randomly split into two subsamples (N = 255 and 233)*.

## Study 2

### Methods

#### Participants

Participants consisted of 50 first-year psychology students from the University of New South Wales, who completed the study in return for course credit. One (1) participant was excluded from the study due to only partial completion of the questionnaires, with 49 participants included in the final analysis. Credit was individually allocated following the completion of each portion of the study. The study received approval from the Human Research Ethics Committee (Psychology) of the University of New South Wales (HREAP: 153–185). Participants constituted 44 females and five males, with a mean age of 19.33 ± 1.26 years (17–24 years). The majority of participants were born in Australia (78%), with mixed ethnicity (51% Asian, 16% mixed, 6% Middle Eastern, 4% Indian, 4% African, and 18% no answer). Up to 61% of participants reported part-time employment, and 76% were single and not in a current relationship. The sample was mostly psychiatrically healthy, with four participants reporting ongoing mental health problems (anxiety or mood disorder), and a further two participants reporting past mental health problems.

#### Measures and procedure

Participants completed the CT scale online, and then a battery of questionnaires to measure different aspects of well-being, and depression/anxiety risk symptoms, as listed below. To limit fatigue, participants were provided a 10-min break as required. Upon completion, participants were debriefed and invited to return for a retest session, which took place 2 weeks after their initial test session. Twenty-five participants returned to complete the second session. During the retest session, participants completed the CT scale again, as well as the COMPAS-W, DASS-21, and mDES scales (Lovibond and Lovibond, 1995; Fredrickson et al., 2003; Gatt et al., 2014). Participants were debriefed upon the study's completion.

1. Composite Well-Being Measures. Composite well-being was measured by the COMPAS-W Wellbeing Scale (Gatt et al., 2014) and the MHC-SF MHC Scale (Keyes, 2009).
2. Subjective Well-Being (SWB) Measures. SWB was measured using the Satisfaction with Life Scale (SWLS) (Diener et al., 1985), WHOQoL Quality of Life Scale (Murphy et al., 2000), and the mDES modified Differential Emotions Scale (Fredrickson et al., 2003).
3. Psychological Well-Being (PWB) Measures. Measures of PWB were taken using Ryff's scales of Psychological Well-being (Springer and Hauser, 2006), the GQ-6 Gratitude questionnaire (Mccullough et al., 2002), and the MAAS Mindful Attentional Awareness scales (Brown and Ryan, 2003).
4. Measures of Depression/Anxiety Risk Symptoms. Participants' depression and anxiety symptom risk levels were measured using the DASS-21 Depression Anxiety Stress Scale (Lovibond and Lovibond, 1995).

#### Analysis

Internal consistency reliability of the CT scale and comparable questionnaires was evaluated using Cronbach alpha. The test-retest reliability of each scale over 2 weeks was evaluated using intra-class correlations. Inter-correlations between the CT subscales at baseline were also considered. Correlations between the CT scale and the well-being/risk symptom questionnaires were observed at the corrected threshold of *p* < 0.0003. We also examined some basic demographic associations with the CT scale, including associations with age, sex, marital, and occupation status.

### Results

#### Sample demographics

The means and standard deviations (SD) of Study 2 participants for all the scales are provided in Table 2.

**Table 2:** Sample means (± Standard Deviations), internal consistency reliability, and test-retest reliability of all measures in Study 2.

**Measure**	**Number of items**	**Means ± SD**	**Internal consistency (*N* = 49): Cronbach alpha (α)**	**Test-retest reliability (*N* = 25): Intra-class Coefficient (ICC)**
**CT SCALE FOR WELL-BEING**				
CT total Well-being	60	74.32 ± 10.78	0.96	0.63
CT family	12	72.17 ± 12.20	0.90	0.70
CT self	12	66.94 ± 14.63	0.87	0.63
CT relationship	12	79.31 ± 11.57	0.88	0.53
CT work	12	74.34 ± 11.59	0.85	0.69
CT community	12	78.83 ± 11.46	0.89	0.77
**COMPOSITE WELL-BEING MEASURES**				
COMPAS-W total wellbeing	26	95.24 ± 12.82	0.91	0.91
COMPAS-W composure	4	14.14 ± 2.92	0.77	0.84
COMPAS-W ownworth	9	32.84 ± 4.54	0.75	0.87
COMPAS-W mastery	6	22.86 ± 3.32	0.79	0.85
COMPAS-W positivity	5	19.37 ± 3.13	0.77	0.87
COMPAS-W achievement	3	10.53 ± 2.28	0.79	0.78
COMPAS-W satisfaction	9	32.29 ± 6.23	0.88	0.85
MHCSF total score	14	46.51 ± 12.17	0.93	–
MHCSF hedonia emotional well-being	3	11.73 ± 2.73	0.88	–
MHCSF eudaimonia social well-being	5	14.71 ± 5.03	0.79	–
MHCSF Eudaimonia psychological well-being	6	20.06 ± 5.69	0.89	–
**SUBJECTIVE WELL-BEING (HEDONIA) MEASURES**				
Satisfaction with life scale (SWLS)	5	24.69 ± 6.25	0.87	–
WHO-QOL physical	7	111.84 ± 16.21	0.77	–
WHO-QOL psychological	6	84.08 ± 16.88	0.85	–
WHO-QOL relationship	3	42.78 ± 8.73	0.67	–
WHO-QOL environment	8	128.82 ± 15.53	0.75	–
mDES positive emotions	10	3.46 ± 0.75	0.91	0.77
mDES negative emotions	10	2.12 ± 0.83	0.91	0.57
**PSYCHOLOGICAL WELL-BEING (EUDAIMONIA) MEASURES**				
Ryff's psychological well-being: autonomy	7	26.98 ± 6.17	0.86	–
Ryff's psychological well-being: environmental mastery	7	26.92 ± 3.98	0.49	–
Ryff's psychological well-being: growth	7	31.45 ± 5.47	0.83	–
Ryff's psychological well-being: positive relations	7	32.22 ± 5.88	0.81	–
Ryff's psychological well-being: purpose	7	29.41 ± 5.01	0.76	–
Ryff's psychological well-being: acceptance	7	26.98 ± 7.27	0.90	–
Gratitude questionnaire (GQ-6)	6	34.29 ± 6.42	0.87	–
Mindful attentional awareness scale (MAAS)	15	3.88 ± 0.85	0.91	–
**DEPRESSION AND ANXIETY RISK SYMPTOMS**				
DASS-21 total score	21	24.12 ± 22.16	0.95	0.89
DASS-21 depression score	7	7.39 ± 8.63	0.92	0.87
DASS-21 anxiety score	7	6.29 ± 6.95	0.82	0.85
DASS-21 stress score	7	10.45 ± 9.00	0.89	0.86

*Study 2 included 49 first-year psychology students from Sydney Australia. Test-retest reliability was assessed over a 2-week period for the scales CT, COMPAS-W, mDES, and DASS-21*.

#### Reliability

Internal consistency reliability estimates (Cronbach alpha) suggested good to strong reliability for the CT total scale and subscales: CT Total Well-being (0.96), CT Family (0.90), CT Self (0.87), CT Relationship (0.88), CT Work (0.85), and CT Community (0.89). The test-retest reliability (ICC) showed mostly moderate to good stability over 2 weeks: CT Total Well-being (0.63), CT Family (0.70), CT Self (0.63), CT Relationship (0.53), CT Work (0.69), and CT Community (0.77). Table 2 provides the internal consistency reliability and test-retest reliability estimates for the CT scale and comparable questionnaires. Inter-correlations between the CT subscales were moderate to strong and all positively correlated (see Supplementary Table 7).

#### Association analyses

The CT Total Well-being scale and the CT subscales demonstrated moderate to strong positive correlations with most of the different well-being questionnaires measuring composite as well as subjective and psychological measurements of well-being (see Table 3). The CT Total Well-being scale and subscales also consistently demonstrated negative correlations with questionnaires measuring negative affect (mDES negative emotions) and depression/anxiety risk symptoms (DASS-21) (see Table 3).

**Table 3:** Correlations between the CT Scales and other Well-being / Risk Symptoms Questionnaires in Study 2 (*N* = 49).

**Measure**	**CT Total well-being**	**CT family**	**CT self**	**CT relationship**	**CT work**	**CT community**
**COMPOSITE WELL-BEING MEASURES**						
COMPAS-W total wellbeing	**0.83**	**0.68**	**0.83**	**0.75**	**0.66**	**0.69**
COMPAS-W composure	**0.65**	**0.52**	**0.69**	**0.58**	**0.56**	0.47
COMPAS-W Ownworth	**0.62**	0.45	**0.62**	**0.53**	**0.55**	**0.53**
COMPAS-W mastery	**0.52**	0.33	**0.52**	0.48	**0.52**	0.42
COMPAS-W positivity	**0.71**	**0.59**	**0.65**	**0.70**	0.49	**0.67**
COMPAS-W achievement	**0.61**	**0.56**	**0.59**	0.47	**0.54**	**0.51**
COMPAS-W satisfaction	**0.76**	**0.65**	**0.80**	**0.72**	**0.54**	**0.59**
MHCSF total Score	**0.72**	**0.70**	**0.69**	**0.59**	**0.51**	**0.65**
MHCSF hedonia emotional well-being	**0.64**	**0.64**	**0.67**	**0.57**	0.36	**0.56**
MHCSF eudaimonia social well-being	**0.72**	**0.70**	**0.66**	**0.55**	**0.58**	**0.67**
MHCSF eudaimonia psychological well-being	**0.59**	**0.56**	**0.58**	**0.50**	0.41	**0.53**
**SUBJECTIVE WELL-BEING (HEDONIA) MEASURES**						
Satisfaction with life scale (SWLS)	**0.68**	**0.68**	**0.70**	**0.60**	0.40	**0.58**
WHO-QOL physical	**0.63**	**0.51**	**0.67**	**0.71**	0.42	0.42
WHO-QOL psychological	**0.76**	**0.70**	**0.84**	**0.68**	**0.54**	**0.53**
WHO-QOL relationship	**0.52**	0.49	**0.52**	**0.54**	0.29	0.42
WHO-QOL environment	0.44	0.40	0.33	0.34	0.37	0.49
mDES positive emotions	**0.63**	**0.67**	**0.59**	0.38	0.46	**0.63**
mDES negative emotions	−**0.53**	−**0.50**	−**0.60**	–0.47	–0.38	–0.31
**PSYCH WELL-BEING (EUDAIMONIA) MEASURES**						
Ryff's psychological well-being: autonomy	0.38	NS	0.48	0.39	NS	NS
Ryff's Psychological Well-being: Environmental Mastery	**0.72**	**0.68**	**0.75**	**0.62**	0.47	**0.58**
Ryff's psychological well-being: growth	**0.58**	**0.54**	**0.55**	**0.51**	0.44	0.49
Ryff's psychological well-being: positive relations	**0.59**	0.49	**0.50**	**0.62**	0.40	**0.56**
Ryff's psychological well-being: purpose	**0.64**	**0.56**	**0.62**	**0.58**	**0.53**	**0.51**
Ryff's psychological well-being: acceptance	**0.72**	**0.64**	**0.80**	**0.66**	0.49	**0.51**
Gratitude questionnaire (GQ-6)	**0.69**	**0.67**	**0.59**	**0.52**	**0.58**	**0.67**
Mindful attentional awareness scale (MAAS)	**0.51**	**0.52**	0.42	0.41	0.47	0.42
**DEPRESSION AND ANXIETY RISK SYMPTOMS**						
DASS-21 total score	−**0.70**	−**0.66**	−**0.74**	−**0.60**	−**0.59**	–0.46
DASS-21 depression score	−**0.72**	−**0.69**	−**0.76**	−**0.56**	−**0.60**	–0.49
DASS-21 anxiety score	−**0.69**	−**0.61**	−**0.73**	−**0.60**	−**0.60**	–0.44
DASS-21 stress score	−**0.52**	–0.48	−**0.53**	–0.47	–0.42	–0.33

*Bolded correlations are significant at corrected threshold of p < 0.0003. Unbolded correlations are significant at uncorrected p < 0.05. NS = not significant (p > 0.05)*.

#### Demographic comparisons

There were no significant associations between age, sex or marital relationship status and mean scores on the CT scales (*p* > 0.05). A significant association was however found between employment status and mean CT Self scores whereby individuals who were employed demonstrated a higher mean score (mean ± SD: 70.22 ± 13.25, *N* = 30) than unemployed individuals (mean ± SD: 61.76 ± 15.55, *N* = 19).

## Discussion

The aim of the current research was to assess the psychometric properties of a new questionnaire that measures the Centeredness Theory of well-being, which is a systems approach to self-actualization. The results from two studies were reported. In Study 1, a psychometric validation study of the original and refined scale was conducted using exploratory and confirmatory factor analytic techniques in 488 adult anonymous online responders. The participants for Study 1 completed the CT scale online from one of 38 countries worldwide, with the largest number of participants originating from Oceania, North America and the United Kingdom. Both males and females completed the form, ranging in age from 20 to 70 years, with most reporting being in a relationship and employed. The analyses aimed to confirm whether the CT domains and sub-domains were structurally sound, and to quantify their contribution to overall centeredness. Exploratory factor analysis results suggested a reduced 60-item solution from the original 108 items. A total of five domains was confirmed (Self, Family, Relationship, Work, Community) with a four-factor solution derived per domain (i.e., 12 items per sub-domain). Confirmatory congeneric models supported the structural integrity of the four-factor sub-domain solution per domain. Acceptable internal consistency reliability of each domain was indicated by the Cronbach Alpha coefficients (majority > 0.70). A larger one-factor congeneric nested model of all five domains was also confirmed, which suggested that the overarching construct of Centeredness to be a common construct across the five domains. In particular, the weighted factor score coefficients suggested that the inner Self domain accounted for 54.40% of the total variance of the common factor Centeredness.

In Study 2, an independent reliability study of the refined 60-item questionnaire was conducted to confirm its internal consistency reliability, test-retest reliability and convergent validity against existing well-being and mental health questionnaires in 49 adults over two time-points (2 weeks apart). Study 2 participants were first-year Psychology students from Sydney Australia, the majority of which were females and were of a younger demographic than Study 1, ranging in age from 17 to 24 years. Unlike Study 1, Study 2 participants were also mostly born in Australia, and the majority reported being single (rather than in a relationship) and with part-time employment. Internal consistency reliability for each of the CT domains was good (all >0.80) in the independent sample, and was comparable to the reliability estimates identified for other well-being questionnaires (e.g., the COMPAS-W and MHCSF composite well-being scales, and relative to other subjective or psychological well-being measures). Test-retest reliability estimates over a 2-week period of the CT subscales was within the moderate to good range, ranging from intra-class coefficient estimates of 0.53 (for the “Relationship” domain) to 0.77 (for the “Community” domain). Similar test-retest reliability estimate ranges were also found for the mDES Positive and Negative Emotion scale (ranging from 0.57 to 0.77), but comparably stronger estimates were evident for other scales (e.g., for COMPAS-W Wellbeing Scale, all > 0.70; and the DASS-21 Depression, Anxiety, Stress Scale, all > 0.8). One reason for the lower test-retest reliability estimates for the CT and mDES measures than the COMPAS-W and DASS-21 measures may be because the former measures are more state-based than the latter measures. It is also possible that the lower estimates (particularly for the Relationship domain) are due to the sample constituting mostly single young adults (76%) that are not in a relationship, and therefore many of these particular items were not relevant to their current situation. Future studies should confirm these effects in a larger and more heterogeneous sample that varies in sex distribution, ethnicity but also other key demographic characteristics such as relationship and occupational status. Inter-correlations between the CT domain subscales were quite strong, supporting the internal structure of the whole scale. Furthermore, correlations between the CT subscales and all the other questionnaires were in the predicted direction, and were evident across both composite measures of well-being and more specific subjective and psychological well-being indices. Future studies could however consider more explicit measures of convergent validity of the different domains by including a more expansive range of measures. For instance, to validate the Work domain, specific measures of work performance could be administered to examine the specificity of this measure with work satisfaction.

Together, these findings support the 60-item CT Scale as a measure of Centeredness of well-being across the five domains of Self, Relationship, Family, Work, and Community. The results suggest that this measure is mostly reliable, although some items may need some further development to ensure stability across samples. The final nested structural model for CT identified in Study 1 and portrayed in Figure 4 provided support for the theory. Consistent with hypotheses, this model first demonstrated significant correlations between the five core domains; that is, between Self and Family, and between Self and Relationship, and so forth. Second, the model demonstrated significant direct paths from each domain to its four sub-domains; for example, between Self and its four sub-domains of Adaptability, Awareness, Contentment and Inspiration, as hypothesized. Finally, the model also identified several cross-loadings between specific domains and other sub-domains. While these specific cross-loadings were suspected may arise they were not initially predicted, and are consistent with the original model because they show how different domains may similarly impact, or be impacted by, the same sub-domain that share a common quality. For instance, Family demonstrated a cross-loading onto the Relationship sub-domain of Connection. This sub-domain measures the level of priority of an individual's relationship with their partner, and therefore it is foreseeable that the level of connection with one's partner would also impact the level of centeredness within the larger Family domain. Thus, overall, centeredness is a set of distinct factors that make an interconnected whole, where balance (or imbalance) within one domain or sub-domain facilitates (or inhibits) balance in the other domains. In addition, in Study 2 we found correlations between CT Total Well-being, CT Self, and the other measures of well-being. While this result would seem to suggest that CT Total Well-being is not more correlated to other measures of mental health and well-being than CT Self, this is also not a surprising effect as many of the existing scales measure well-being at the “self” level alone. Consistent with this, the other scales of mental health and well-being demonstrate less consistent correlations with the other CT domains (CT Family, CT Relationship, CT Work, and CT Community), highlighting some differentiation at this level of measurement.

With some developments, the CT scale could be used to measure levels of centeredness in the general population. For example, the theoretical model should be tested further in different cultural contexts and population demographics (e.g., by age, sex, and employment status). While it is possible that the scale may only apply to specific countries (e.g., western, industrialized, educated, and/or democratic) it is predicted that CT may have cross-cultural application because every individual has meaningful goals that relate to the self (Sheldon et al., 2004), and it could be argued that the domains of Family, Relationship, Community, and Work are universal goals to the human experience. Yet, one area for further study is to determine whether centeredness may balloon differently between the domains and sub-domains in cultures that are individualist and collectivist. While the final nested model in Figure 4 suggests some cross-cultural integrity in direct paths, correlations and cross-loadings across the various participants currently examined, further testing is required in Asia, South America, and Africa and the items translated. Another area for further study is the universality of the core life domains and whether each domain is applicable to every person at each stage of their life. For instance, one could consider the effect of life span on CT and whether the Work domain is applicable throughout the different ages as a person shifts from schooling to paid work and then later life unpaid work, including volunteer or caregiver roles. We predict that the Work domain can operate across these dimensions, whereby the items may need to be modified to include (for instance) the school experience with some minor prescriptive modifications to the item wording. The same rationale could be applied to the other domains, such as the Relationship domain for adolescence to young adulthood, whereby “close friend” or “best friend” could be used to replace the word “partner” for these particular item sets (Adams et al., 2011; Kochel et al., 2012).

Another possible future application is the use of algorithmic models to test how changing parameters in one domain (e.g., Relationship) could impact the other domains and the whole system. Equipped with a set of drivers and levers, for instance, interventions could be developed and targeted on both the domain and sub-domain levels to enhance an individual's centeredness. A good instrument to facilitate this approach may be a driver tree. The driver tree concept was developed by F Donaldson Brown at Dupont de Nemours and Co.^1^ (Flesher and Previts, 2013), and it is feasible that it could be applied to well-being scales, like CT, that take a systems-approach to well-being. A well-being driver tree (WBDT) based on CT could identify the domain and sub-domains that impact well-being negatively, highlight domains for improvement and drive intervention programs to promote overall well-being.

The five life domains and twenty sub-domains would make the operational drivers of the tree. An interconnected-relationships algorithm could then be introduced to determine the span of control across the five life domains and twenty sub-domains, using information derived from Figure 4, which demonstrate how each sub-domain and domain of CT affects the whole system. Equipped with a set of operational drivers, both the causes of reduced well-being and levers to enhance well-being could be identified on both the domain and sub-domain levels. Interventions to promote well-being could be made prescriptive based on the WBDT. For example, reduced well-being could be targeted at the sub-domain level and interventions accurately selected to pinpoint the cause. For instance, mindfulness could be tested as an intervention for individuals rating lower on the sub-domain “awareness” (within the domain Self) (Hofmann et al., 2010). Similarly, forgiveness therapy (Enright and Fitzgibbons, 2015) could be tested and applied for individuals rating low on the sub-domain Receptiveness (within the domain Family). The Gottmann Method (Gottmann, 1994) could be tested for utility in those individuals scoring lower in one or more sub-domains of Relationship. The WBDT could be used to compare the value of alternative strategies and to choose the best strategy that maximizes CT Total Well-Being.

In conclusion, this study describes the development and psychometric validation of a new 60-item scale of centeredness that may be used to measure well-being across different life domains. Results from two studies were described and evidence shown toward the confirmatory factorial structure of the CT scale at both the domain and whole scale level, as well as psychometric evidence to support its sub-domain efficacy. While there may be scope for some specific item improvement, it would be important to confirm its reliability and validity in larger and more heterogeneous samples.

## Ethics statement

Study 1 included participants who were anonymous online users. All respondents agreed to supply their responses, but maintain anonymity for use in scientific research. This study was exempt from ethical review per national guidelines as the data was already existing prior to the study, and was low risk and de-identified. Study 2 received approval from the Human Research Ethics Committee (Psychology) of the University of New South Wales (HREAP: 153–185). Participants were first-year psychology students from the University of New South Wales, who provided informed consent and completed the study in return for course credit.

## Author contributions

ZB-J developed Centeredness Theory. PC conducted the EFA and CFA analysis of the CT Scale and write-up for Study 1 with ZB-J. WY collected data for Study 2 and completed data entry and scoring as part of his third-year psychology research internship. JG supervised WY and the conduct of Study 2 and conducted the Study 2 analysis and write-up. All authors contributed toward manuscript preparation and final editing.

### Conflict of interest statement

ZB-J is CEO of MAP Corporation Pte., Ltd. and will receive income from MAP Corporation Pte., Ltd. MAP Corporation Pte., Ltd. developed and owns the MAP technology. MAP is offered as not-for-profit product for individuals and for-profit for enterprises with financial interest for ZB-J as stockholder. PC is Director of Sciens Pty. Ltd., with 50% ownership in the company. JG is a stockholder in MAP Corporation Pte., Ltd. No authors received payment from MAP Corporation Pte., Ltd. for this work. WY declares that the research was conducted in the absence of any commercial or financial relationships that could be construed as a potential conflict of interest.

## Footnotes

^1^To date the driver tree concept has been used widely to measure and drive productivity and has become a national standard (Flesher and Previts, 2013). The tree is used to identify factors (or drivers) that affect productivity and how they interconnect. Driver trees can identify weaknesses, highlight areas for improvement, and can be used to benchmark and compare alternative strategies. Because a driver tree is modeled on a relationship of factors, a systems-approach to well-being could be a good candidate for this type of modeling.
