# Corrigendum: Centeredness Theory: Understanding and Measuring Well-Being Across Core Life Domains

**DOI:** 10.3389/fpsyg.2018.01648

**Published:** 2018-09-07

**Authors:** Zephyr T. Bloch-Jorgensen, Patrick J. Cilione, William W. H. Yeung, Justine M. Gatt

**Affiliations:** ^1^MAP Corp. Pte. Ltd., Atlanta, Atlanta, GA, United States; ^2^MAP Corp. Pte. Ltd., Singapore, Singapore; ^3^Sciens Pty. Ltd., Bundoora, VIC, Australia; ^4^School of Psychology, University of New South Wales, Sydney, NSW, Australia; ^5^Neuroscience Research Australia, Randwick, NSW, Australia

**Keywords:** wellbeing, flourishing, mental health, mindfulness, self-actualization, goal-setting, COMPAS-W

In the original article, there was a mistake in *Supplementary Figure 1* as published. The Supplementary Figure that was submitted at the time of publishing was mislabeled. The corrected *Supplementary Figure 1* appears below. The authors apologize for this error and state that this does not change the scientific conclusions of the article in any way.

**Supplementary Figure 1:**
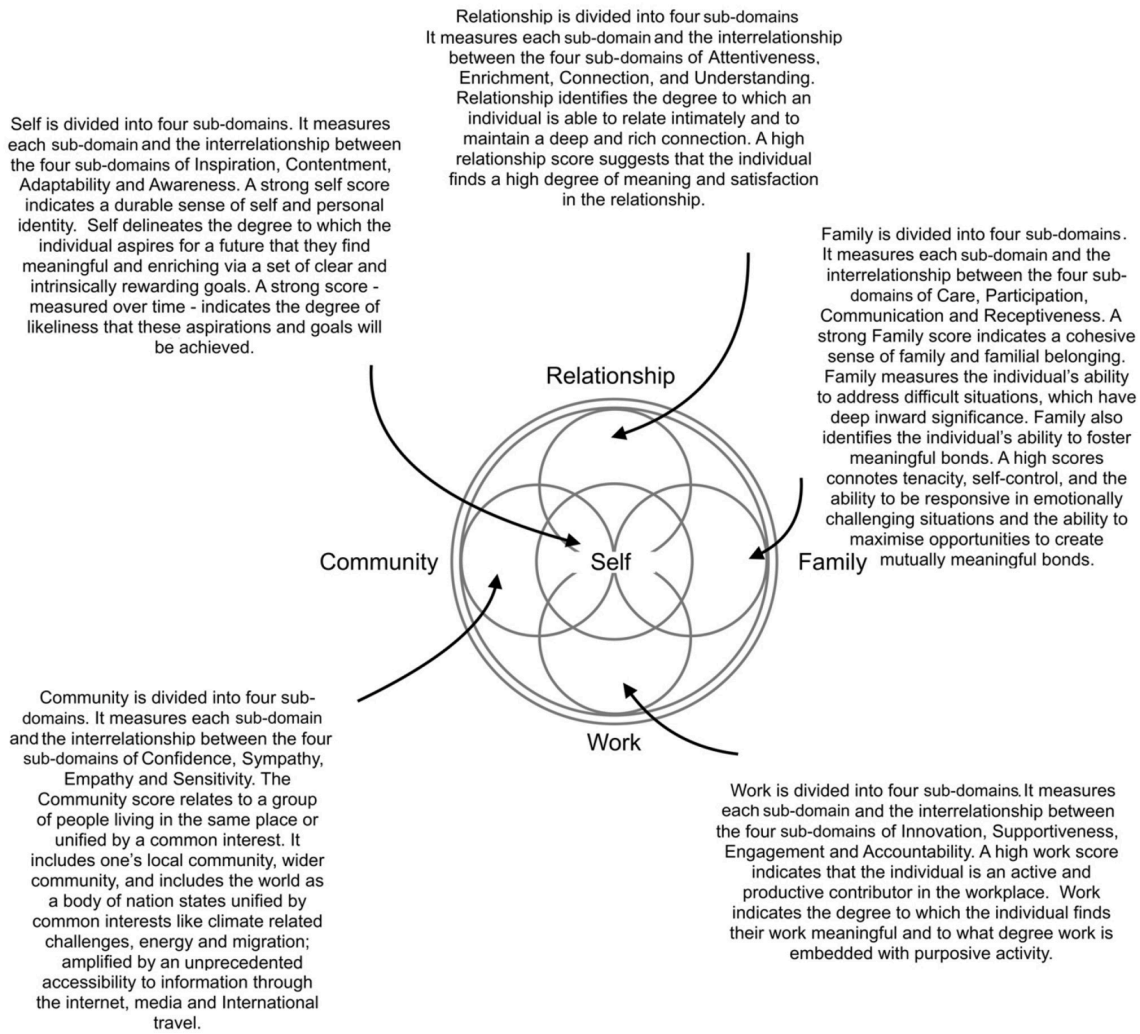
Domain Descriptions.

The original article has been updated.

## Conflict of interest statement

ZB-J is CEO of MAP Corporation Pte., Ltd. and will receive income from MAP Corporation Pte., Ltd. MAP Corporation Pte., Ltd. developed and owns the MAP technology. MAP is offered as not-for-profit product for individuals and for-profit for enterprises with financial interest for ZB-J as stockholder. PC is Director of Sciens Pty. Ltd., with 50% ownership in the company. JG is a stockholder in MAP Corporation Pte., Ltd. No authors received payment from MAP Corporation Pte., Ltd. for this work. WY declares that the research was conducted in the absence of any commercial or financial relationships that could be construed as a potential conflict of interest.

