# Supplementary material for: Centeredness Theory: Understanding and Measuring Well-Being Across Core Life Domains
Source: Front Psychol. 2018 May 1;9:610. doi: 10.3389/fpsyg.2018.00610 (PMC5938389; doi:10.3389/fpsyg.2018.00610)
Supplement: Supplementary file 1 [file Table_1.DOCX]

**Supplementary Table 1: Original Centeredness Theory Scale Item Structure**

| Domain | Sub-domain | Sub-domain total | Domain total |
| --- | --- | --- | --- |
| Family | Care  Communication  Participation  Receptiveness | 4 | 19 |
|  |  | 4 |  |
|  |  | 5 |  |
|  |  | 6 |  |
| Self | Adaptability  Awareness  Contentment  Inspiration | 3 | 19 |
|  |  | 4 |  |
|  |  | 7 |  |
|  |  | 5 |  |
| Relationship | Enrichment  Attentiveness  Connection  Understanding | 8 | 22 |
|  |  | 7 |  |
|  |  | 3 |  |
|  |  | 4 |  |
| Work | Accountability  Innovation  Engagement  Supportiveness | 4 | 22 |
|  |  | 4 |  |
|  |  | 5 |  |
|  |  | 9 |  |
| Community | Confidence  Empathy  Sensitivity  Sympathy | 7 | 18 |
|  |  | 3 |  |
|  |  | 4 |  |
|  |  | 4 |  |
