# Supplementary material for: Centeredness Theory: Understanding and Measuring Well-Being Across Core Life Domains
Source: Front Psychol. 2018 May 1;9:610. doi: 10.3389/fpsyg.2018.00610 (PMC5938389; doi:10.3389/fpsyg.2018.00610)
Supplement: Supplementary file 2 [file Table_2.DOCX]

**Supplementary Table 2: Guidelines for determining model fit**

| Fit Index | Acceptable Threshold Levels | Description |
| --- | --- | --- |
| Absolute Fit Indices |  |  |
| Chi-Square | Low Chi-Square relative to degrees of freedom with insignificant p value (p > 0.05) |  |
| RMSEA | Values less than 0.07 (Steiger, 2007) | Has a known distribution. Favours parsimony. Values less than 0.03 represent excellent fit. |
| SRMR | SRMR less than 0.08 (Hu & Bentler, 1999) | Standardised version of the RMR. Easier to interpret due to its standardised nature. |
| Incremental Fit Indices |  |  |
| NNFI | Values greater than 0.95 | Non-normed, values can fall outside the 0-1 range. Favours parsimony. Performs well in simulation studies (McDonald & Ho, 2002; Sharma, Mukherjee, Kumar, & Dillon, 2005) |
| CFI | Values greater than 0.95 | Normed, 0-1 range. |
| Parsimonious Fit Index |  |  |
| PNFI | No thresholds set | Adjustment to normed fit index (NFI), that penalise models that are less parsimonious. |

**References**

Hu, L. T., & Bentler, P. M. (1999). Cutoff criteria for fit indices in covariance structure analysis: Conventional criteria versus new alternatives. *Structural Equation Modeling, 6*(1), 1-55.

McDonald, R. P., & Ho, M.-H. R. (2002). Principles and practice in reporting statistical equation analyses. *Psychological Methods, 7*(1), 64-82.

Sharma, S., Mukherjee, S., Kumar, A., & Dillon, W. R. (2005). A simulation study to investigate the use of cutoff values for assessing model fit in covariance structure models. *Journal of Business Research, 58*(1), 935-943.

Steiger, J. H. (2007). Understanding the limitations of global fit assessment in structural equation modeling. *Personality and Individual Differences, 42*(5), 893-898.
