# Supplementary material for: Centeredness Theory: Understanding and Measuring Well-Being Across Core Life Domains
Source: Front Psychol. 2018 May 1;9:610. doi: 10.3389/fpsyg.2018.00610 (PMC5938389; doi:10.3389/fpsyg.2018.00610)
Supplement: Supplementary file 4 [file Table_4.DOCX]

**Supplementary Table 4: CT Questionnaire Items and Factor Loadings for each Domain**

Each column shows the results for the first subsample (N_1_ = 255), and the results in the second subsample (N_2_ = 233) are presented in parentheses.

| Domain | Sub-domains | Item (item number) | Factor loadings | % Variance | Cronbach alpha |
| --- | --- | --- | --- | --- | --- |
| Family | Care (fa1) | Do you know what it means to love a member of your family? (1) | .80 (.77) | 14.85 (14.21) | .79 (.83) |
|  |  | Do you listen to your family? (2) | .51 (.39) |  |  |
|  |  | Do you instil love within your family? (3) | .71 (.46) |  |  |
|  | Communication (fa2) | Do you share your feelings with your family? (4) | .87 (.88) | 21.27 (24.26) | .86 (.88) |
|  |  | Do you tell your family how you feel? (5) | .89 (.88) |  |  |
|  |  | When you feel uncomfortable in a situation with your family do you stay open and receptive? (6) | .61 (.67) |  |  |
|  | Participation (fa3) | Do you know what you would like to experience as a member of your family? (The word “family” means the family you were raised in AND the family you may now be responsible for.) (7) | .82 (.81) | 21.06 (24.68) | .87 (.90) |
|  |  | Do you know what you would like to contribute as a member of your family? (8) | .82 (.84) |  |  |
|  |  | Do you know what you want to accomplish as a family? (9) | .78 (.86) |  |  |
|  | Receptiveness (fa4) | Is forgiveness important to love a member of your family? (10) | .68 (.44) | 17.51 (14.78) | .70 (.73) |
|  |  | Do you believe that each member of your family, including your parents, have the right to make mistakes? (11) | .81 (.83) |  |  |
|  |  | Do you accept that members of your family will have a plan for their life that is likely different to your own? (12) | .73 (.81) |  |  |

| Domain | Sub-domains | Item | Factor loadings | % Variance | Cronbach alpha |
| --- | --- | --- | --- | --- | --- |
| Self | Adaptability (se1) | Do you believe that a positive attitude creates opportunities? (13) | .59 (.39) | 15.60 (13.44) | .69 (.73) |
|  |  | Do you make the best of every difficult situation? (14) | .68 (.68) |  |  |
|  |  | Do you welcome change? (15) | .80 (.88) |  |  |
|  | Awareness (se2) | Do you acknowledge to yourself how you feel? (16) | .75 (.76) | 16.99 (17.92) | .73 (.77) |
|  |  | When you feel insecure do you acknowledge this to yourself? (17) | .79 (.86) |  |  |
|  |  | Do you ever acknowledge your fear? (18) | .79 (.73) |  |  |
|  | Contentment (se3) | Do you feel satisfied with who you are? (19) | .88 (.86) | 20.81 (21.98) | .88 (.89) |
|  |  | Are you content with who you have become? (20) | .86 (.83) |  |  |
|  |  | Are you content? (21) | .77 (.84) |  |  |
|  | Inspiration (se4) | Do you think that you have the ability to lead a life that inspires you? (22) | .67 (.72) | 17.53 (18.77) | .80 (.74) |
|  |  | Do you think about your life and explore what you can do to make it more rewarding? (23) | .74 (.79) |  |  |
|  |  | Do you have a clear idea or vision of the life that you would like to lead? For example: The kind of friendships you’d like to have, the type of work you’d like to do, the citizen you’d like to be. (24) | .81 (.56) |  |  |

| Domain | Sub-domains | Item | Factor loadings | % Variance | Cronbach alpha |
| --- | --- | --- | --- | --- | --- |
| Relationship | Enrichment (re1) | Do you feel enriched by your relationship? (25) | .83 (.81) | 23.76 (22.16) | .90 (.90) |
|  |  | Do you see your relationship as an opportunity to achieve a more rewarding life? (26) | .86 (.87) |  |  |
|  |  | Do you believe that your relationship has a purpose? For example: a relationship can give you the opportunity to love, to enable each other’s passions, and to see the world more deeply through a second outlook. (27) | .78 (.80) |  |  |
|  | Attentiveness (re2) | Does your partner think that you are supportive? (28) | .71 (.68) | 23.46 (18.84) | .88 (.80) |
|  |  | Do you listen to your partner? (29) | .79 (.75) |  |  |
|  |  | Does your partner think you listen? (30) | .85 (.86) |  |  |
|  | Connection (re3) | Do you make time for your partner despite the pressure of competing obligations? (31) | .52 (.71) | 11.88(13.57) | .80 (.73) |
|  |  | Does your relationship take precedence over your work? (32) | .90 (.90) |  |  |
|  |  | Do you act in ways to demonstrate the love that you have for your partner on a daily basis? (33) | .25 (.37) |  |  |
|  | Understanding (re4) | Do you tell your partner how you feel about them? (34) | .87 (.89) | 24.19 (24.24) | .92 (.92) |
|  |  | Do you show your partner how you feel about them? (35) | .74 (.79) |  |  |
|  |  | Do you tell your partner how you feel? (36) | .86 (.83) |  |  |

| Domain | Sub-domains | Item | Factor loadings | % Variance | Cronbach alpha |
| --- | --- | --- | --- | --- | --- |
| Work | Engagement (wo1) | Are you happy with the pace at which you are achieving your professional goals? (37) | .71 (.82) | 19.85 (16.15) | .75 (.67) |
|  |  | When you make a mistake, do you see it as a positive? (38) | .82 (.58) |  |  |
|  |  | Each day, do you perform in a way that is consistent with your goals? (39) | .66 (.76) |  |  |
|  | Innovation (wo2) | Can the systems that you use at work, the products you supply, and the services you provide be improved? (40) | .88 (.81) | 13.18 (16.17) | .69 (.72) |
|  |  | Do you enthusiastically question how you work and ask how it can be improved? (41) | .32 (.61) |  |  |
|  |  | Do you ever think about how you can improve your service or product? (42) | .62 (.62) |  |  |
|  | Accountability (wo3) | Do you regard your work as one of the ways to fulfil your life's purpose? (43) | .85 (.82) | 17.70 (19.24) | .73 (.75) |
|  |  | Do you believe that how your organization carries out its function affects the community? (44) | .56 (.74) |  |  |
|  |  | Is it personally important to you that the product or service you supply improves the quality of life of your customers? (45) | .55 (.67) |  |  |
|  | Supportiveness (wo4) | Do you listen to your colleagues? (46) | .76 (.76) | 20.05 (17.58) | .73 (.76) |
|  |  | Are you mindful that your attitude affects others? (47) | .69 (.79) |  |  |
|  |  | Do you believe that a shared purpose in your workplace is valuable? (48) | .73 (.73) |  |  |

| Domain | Sub-domains | Item | Factor loadings | % Variance | Cronbach alpha |
| --- | --- | --- | --- | --- | --- |
| Community | Confidence (co1) | Do you stay positive throughout the day? (49) | .82 (.81) | 16.07 (17.64) | .69 (.69) |
|  |  | Do you feel connected to the people around you? (50) | .81 (.77) |  |  |
|  |  | Do you have a clear idea or vision of the kind of world that you would like to live in? (51) | .41 (.62) |  |  |
|  | Sympathy (co2) | Do you think that understanding another culture is useful? (52) | .70 (.77) | 17.88 (20.19) | .71 (.75) |
|  |  | Do you believe that everyday people, not just people in the public eye, can make a real difference? (53) | .75 (.75) |  |  |
|  |  | Do you believe that a few words of support can change another person’s day? (54) | .59 (.64) |  |  |
|  | Empathy (co3) | Do you treat people of all cultures as equal? (55) | .77 (.88) | 15.33 (14.48) | .70 (.68) |
|  |  | Do you treat people of all social classes as equal? (56) | .46 (.05) |  |  |
|  |  | If you saw a person in the street in need of help, would you assist? (57) | .80 (.75) |  |  |
|  | Sensitivity (co4) | Are you compassionate? (58) | .70 (.52) | 15.86 (15.64) | .63 (.75) |
|  |  | Are you mindful about how you interact with people in everyday situations, like a train station, or talking to a person in a call center? (59) | .69 (.78) |  |  |
|  |  | When you catch yourself judging a person, do you stop? (60) | -.03 (.83) |  |  |
