# Supplementary material for: Centeredness Theory: Understanding and Measuring Well-Being Across Core Life Domains
Source: Front Psychol. 2018 May 1;9:610. doi: 10.3389/fpsyg.2018.00610 (PMC5938389; doi:10.3389/fpsyg.2018.00610)
Supplement: Supplementary file 5 [file Table_5.DOCX]

**Supplementary Table 5: Factor-score Regression Weights and Related Composite Scale Parameters**

| Scale | N^1^ | TD^2^ | Item Weights^3^ | | | | Cronbach alpha^4^ |
| --- | --- | --- | --- | --- | --- | --- | --- |
| Family | 488 |  | fa1 | fa2 | fa3 | fa4 | .830 |
|  |  |  | .325 | .132 | .121 | .080 |  |
|  |  |  | .481 | .207 | .190 | .122 |  |
| Self | 488 |  | se1 | se2 | se3 | se4 | .789 |
|  |  |  | .239 | .088 | .137 | .245 |  |
|  |  |  | .337 | .124 | .194 | .345 |  |
| Relationship | 488 | 2,1 | re1 | re2 | re3 | re4 | .867 |
|  |  |  | .104 | .152 | .178 | .202 |  |
|  |  |  | .163 | .240 | .280 | .317 |  |
| Work | 488 |  | wo1 | wo2 | wo3 | wo4 | .800 |
|  |  |  | .119 | .181 | .229 | .239 |  |
|  |  |  | .155 | .235 | .299 | .311 |  |
| Community | 488 | 3,2 | co1 | co2 | co3 | co4 | .803 |
|  |  |  | .150 | .189 | .151 | .373 |  |
|  |  |  | .174 | .219 | .175 | .432 |  |

^1^ N = the number of cases with complete data

^2^ TD indicates correlated error variance estimates, computed on substantive grounds

^3^ The first row for each scale are the raw factor scores, and the second row are the proportionally weighted factor score coefficients

^4^ The most common measure of reliability is Cronbach Alpha. Cronbach Alpha assesses the internal consistency of the items, that is, the degree to which a set of items are related as a group; and are applied for comparative purposes where acceptable measures are greater than 0.7 as detailed by (Tavakol & Dennick, 2011).

**References**

Tavakol, M., & Dennick, R. (2011). Making sense of cronbach's alpha. *International Journal of Medical Education, 2*, 53-55.
