# Supplementary material for: Centeredness Theory: Understanding and Measuring Well-Being Across Core Life Domains
Source: Front Psychol. 2018 May 1;9:610. doi: 10.3389/fpsyg.2018.00610 (PMC5938389; doi:10.3389/fpsyg.2018.00610)
Supplement: Supplementary file 6 [file Table_6.DOCX]

**Supplementary Table 6: Factor-score Regression Weights and Related Composite Scale Parameters**

| **Scale** | **N^1^** | **TD^2^** | **Item Weights^3^** | | | | | **Cronbach alpha^4^** |
| --- | --- | --- | --- | --- | --- | --- | --- | --- |
| **Centeredness** | 488 | 3,1  5,1  5,3  5,4 | Family | Self | Relationship | Work | Community | .856 |
|  |  |  | .124 | .456 | .039 | .191 | .028 |  |
|  |  |  | .147 | .544 | .047 | .228 | .034 |  |

^1^ N = the number of cases with complete data

^2^ TD indicates correlated error variance estimates, computed on substantive grounds

^3^ The first row for each scale are the raw factor scores, and the second row are the proportionally weighted factor score coefficients

^4^ The most common measure of reliability is Cronbach Alpha. Cronbach Alpha assesses the internal consistency of the items, that is, the degree to which a set of items are related as a group; and are applied for comparative purposes where acceptable measures are greater than 0.7 as detailed by (Tavakol & Dennick, 2011).
