# Supplementary material for: Centeredness Theory: Understanding and Measuring Well-Being Across Core Life Domains
Source: Front Psychol. 2018 May 1;9:610. doi: 10.3389/fpsyg.2018.00610 (PMC5938389; doi:10.3389/fpsyg.2018.00610)
Supplement: Supplementary file 7 [file Table_7.DOCX]

**Supplementary Table 7.** Inter-correlations between the CT Scales in Study 1 (subsamples 1 and 2) and Study 2 participants (all *p* < .0001)

| **Measure** | **Total Well-being** | **Family** | **Self** | **Relationship** | **Work** | **Community** |
| --- | --- | --- | --- | --- | --- | --- |
| **Study 1 (subsample 1, N = 255)** | |  |  |  |  |  |
| CT Total Well-being  CT Family  CT Self  CT Relationship  CT Work  CT Community | - | .92  - | .92  .81  - | .85  .74  .71  - | .85  .70  .79  .54  - | .90  .84  .72  .73  .77  - |
| **Study 1 (subsample 2, N = 233)** | |  |  |  |  |  |
| CT Total Well-being  CT Family  CT Self  CT Relationship  CT Work  CT Community | - | .93  - | .92  .83  - | .83  .73  .67  - | .86  .72  .79  .54  - | .90  .83  .71  .73  .80  - |
| **Study 2 (N = 49)** |  |  |  |  |  |  |
| CT Total Well-being | - | .89 | .90 | .84 | .87 | .89 |
| CT Family  CT Self  CT Relationship  CT Work  CT Community |  | - | .79  - | .63  .72  - | .68  .73  .62  - | .76  .65  .72  .78  - |

*Note.* Study 1 included 488 anonymous online adult respondents across 38 countries randomly split into two subsamples (N=255 and 233), and Study 2 included 49 first-year psychology students from Sydney Australia.
