# Supplementary figures and images for: Centeredness Theory: Understanding and Measuring Well-Being Across Core Life Domains
Source: Front Psychol. 2018 May 1;9:610. doi: 10.3389/fpsyg.2018.00610 (PMC5938389; doi:10.3389/fpsyg.2018.00610)

## Supplementary Figure 1: Domain Descriptions

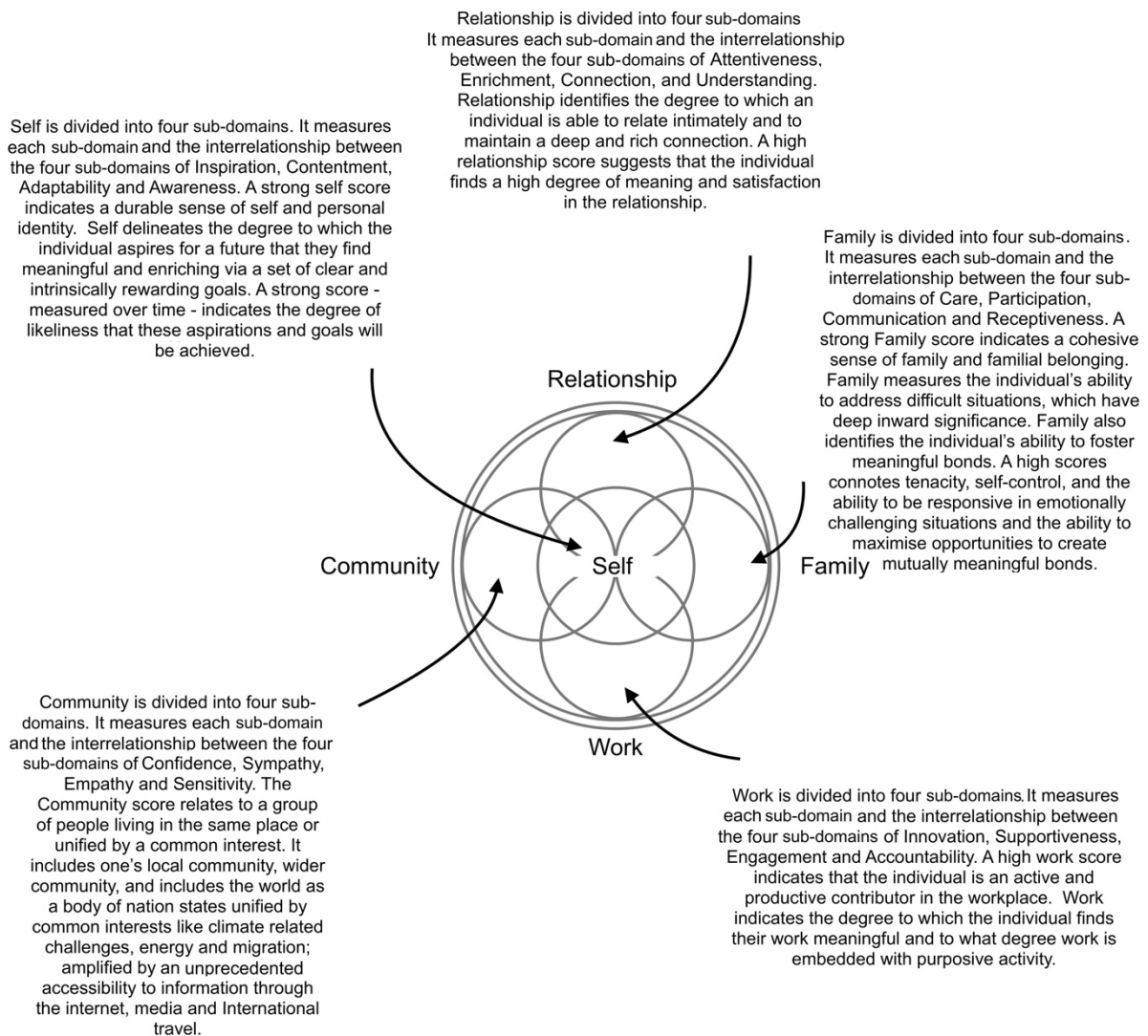

Supplement: Supplementary file 8 [file Image_1.pdf]

## Supplementary Figure 2: Sub-Domain Descriptions

### Self

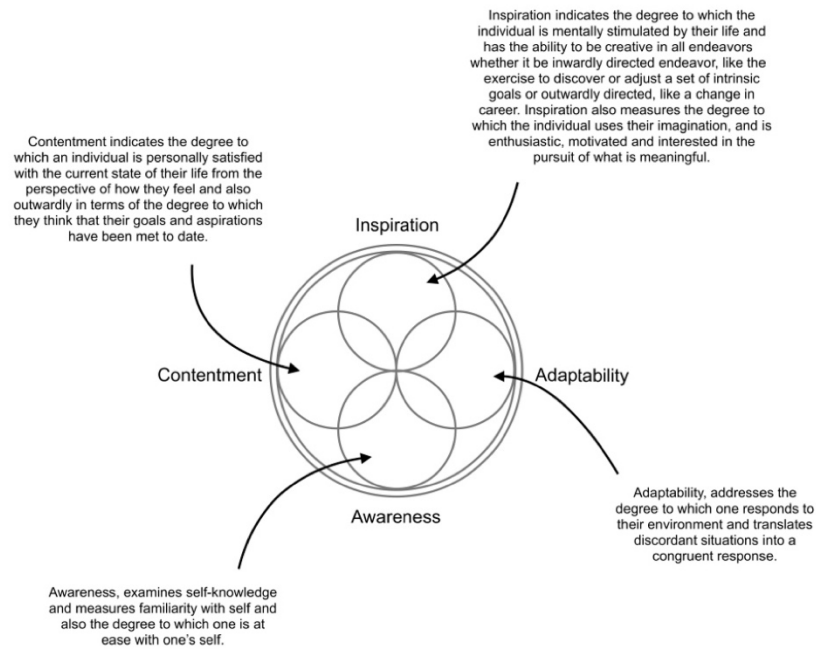

### Relationship

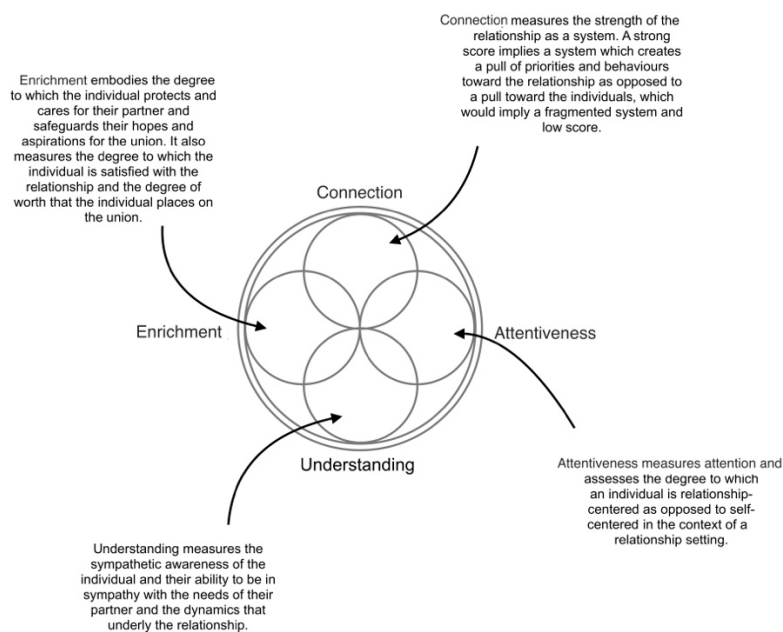

## Community

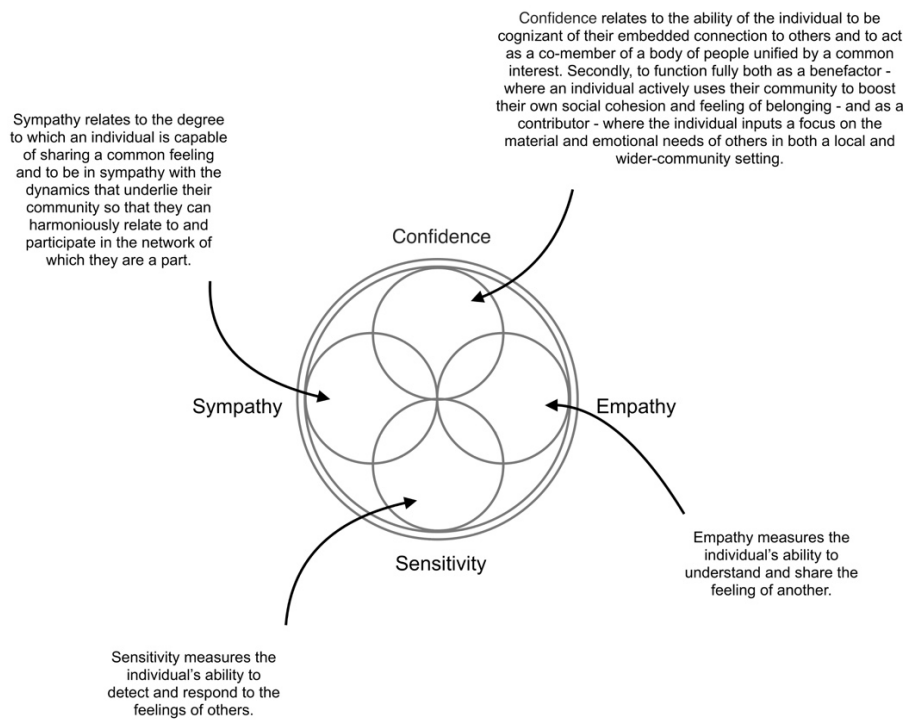

## Family

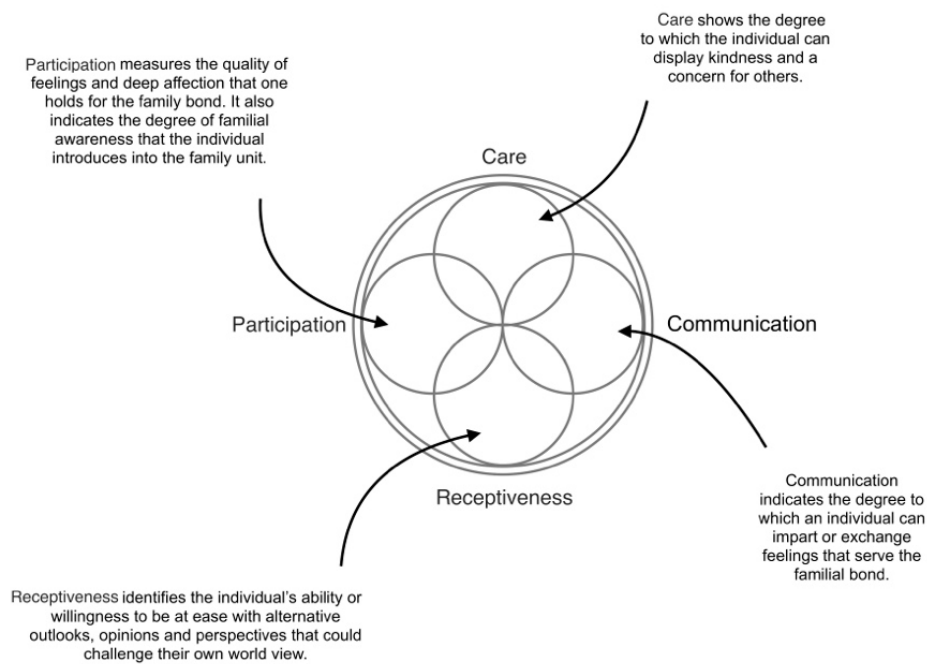

**Work**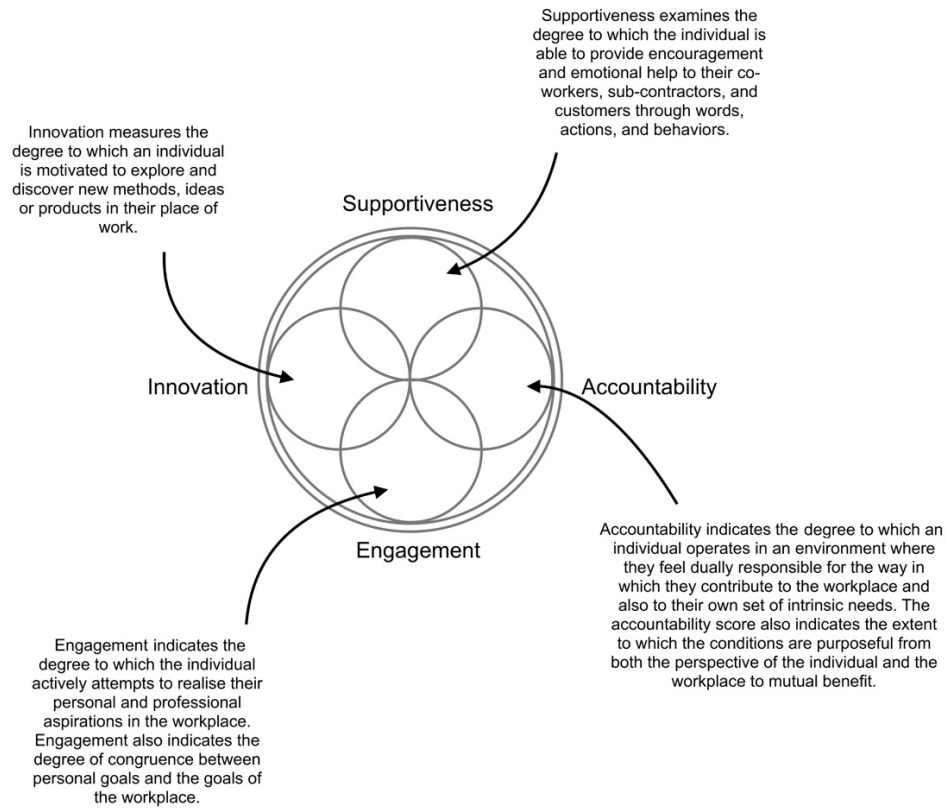

Supplement: Supplementary file 9 [file Image_2.pdf]

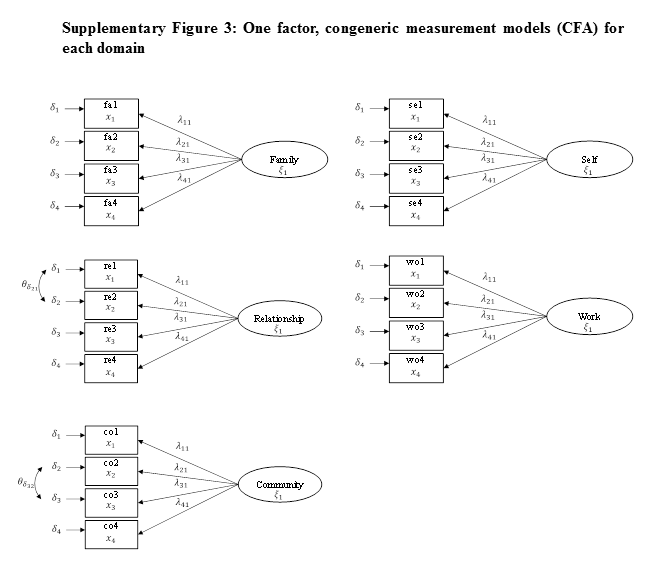

Supplement: Supplementary file 10 [file Image_3.TIF]

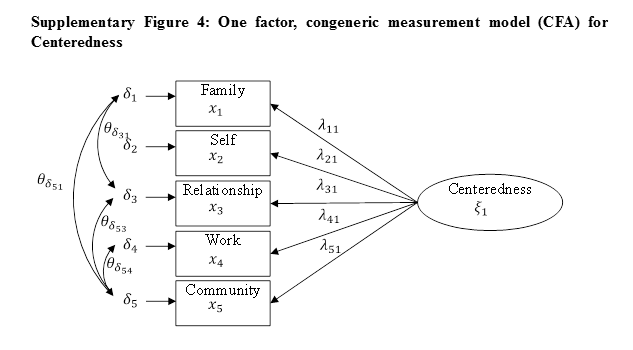

Supplement: Supplementary file 11 [file Image_4.TIF]
